# Determination of Fracture Energy of Early Age Concrete through a Uniaxial Tensile Test on an Un-Notched Specimen

**DOI:** 10.3390/ma13030496

**Published:** 2020-01-21

**Authors:** Dongya Ren, Lambert Houben

**Affiliations:** 1 School of Civil Engineering, Southwest Jiaotong University, Chengdu 610031, China; 2 Highway Engineering Key Laboratory of Sichuan Province, Southwest Jiaotong University, Chengdu 610031, China; 3 Faculty of Civil Engineering and Geoscience, Delft University of Technology, 2628CN Delft, The Netherlands; L.J.M.Houben@tudelft.nl

**Keywords:** early age concrete, fracture energy, uniaxial tensile test, un-notched specimen

## Abstract

Unlike the notched specimens for conventional concrete fracture tests, this paper introduces a deformation-controlled uniaxial tensile test on an un-notched specimen. The surface of the dog bone-shaped specimen is a second order parabolic curve, and the gradual change in the specimen shape does not lead to extreme stress concentrations. Another significant feature of the tension test set-up is that it is built with three hinges, to accommodate the alignment of the specimens. The specimen preparation, test conditions, and the tension test set-up are explained in detail. The fracture energy of the concrete is determined by the obtained complete softening curves. The fracture energy is found to increase with age, going towards a horizontal asymptote as concrete hardened in a tested age range of 1 day to 90 days. Moreover, the rate of development of the fracture energy was found to be higher when compared to tensile strength and stiffness.

## 1. Introduction

The tensile property of concrete at a very early age, in this case as early as the time of the initial setting of the concrete, is essential for the study of cracking control. However, there is very limited relevant information available, especially on the direct measurement of the fracture energy or the complete tensile softening curves for early age concrete. This is mainly due to practical problems, for instance, very early age concrete specimens with a low degree of hydration cannot yet support their own weight without applying any loads [1,2]. Fracture properties, i.e., fracture toughness or fracture energy, have been reported through various types of indirect tensile tests: three-point bending tests [3,4,5], wedge splitting tests [6,7,8], and split tension tests [9,10]. Prismatic or cylindrical specimens with notches are commonly used in the above mentioned fracture energy tests. Adopting a notch in the specimen in the determination of its concrete fracture property has a few drawbacks. Firstly, stress concentrations around the notch are quite large and a significant deviation occurs from the pure uniaxial tensile state of stress [11,12,13]. Moreover, early age concrete is still weak and easily damaged when trying to make a sawcut [9,13]. Cylindrical un-notched specimens introduce the problem that it is unknown where the crack will initiate, which means elaborate instrumentation is needed to control the test and measure the deformation response. Alternatives are so-called “dog-bone” or parabolic-shaped specimens, which localize the crack in the area with the smallest diameter.

This paper introduces a deformation controlled uniaxial tensile test on un-notched specimens. The surface of the dog bone-shaped specimen is a second order parabolic curve, and the gradual change in the specimen shape does not lead to extreme stress concentrations. Another significant feature of the tension test set-up is that it is built with three hinges to accommodate the alignment of the specimens. The specimen preparation, test conditions, and the tension test set-up will be explained in detail. Based on the measured complete softening curves through a uniaxial tensile test on an un-notched specimen, some preliminary results concerning the development of fracture energy of early age concrete will be presented.

## 2. Materials and Methods

### 2.1. Specimen Preparation

**Figure 1:**
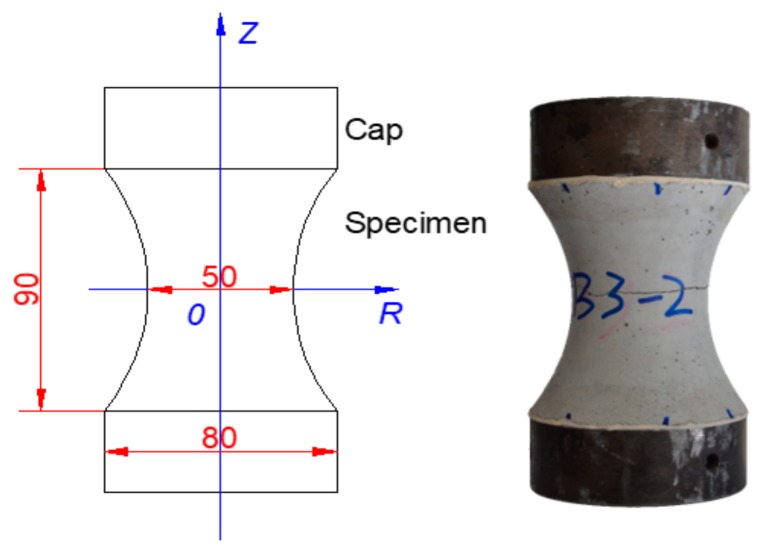
The concrete specimen shape and dimension, in mm.

As shown in Figure 1, the specimen surface is a second order or parabolic curve that has a constant curvature along the entire height of the specimen, therefore reducing the stress concentration. The specimen has its largest diameter of 80 mm at the ends, and it is gradually reduced to 50 mm at the center of the specimen. The nearly 60% reduction of the cross-section area is large enough to initiate the crack at the center and to minimize the boundary effects due to the restrained contraction at the glued end caps. For the typical mixtures applied in two-lift concrete pavements in Belgium, the maximum aggregate size $d_{max}$ of the top layer is 6.3 mm, while it is 32 mm for the bottom layer. Normally, the smallest dimension of the specimen should be taken at least 3 times $d_{max}$ to minimize the boundary effects. Therefore, an aggregate size larger than 16 mm of the bottom layer of concrete is removed from the mixture, while the water/cement ratio, cement content, and sand ratio remain the same as in the original mixture. The gradation is adjusted according to the Fuller curve. The details of the concrete mixtures applied on the motorways E17 (one-lift concrete pavement, maximum grain size 20 mm) and E313 in Belgium can be found in Table 1. CEM III/A 42.5 N/LA is used in the present study. In the subsequent analysis, the concrete specimens with fine aggregate (maximum size 6.3 mm) and coarse aggregate (maximum size 16.0 mm) are called specimen type 6.3 and type 16, respectively.

**Figure 2:**
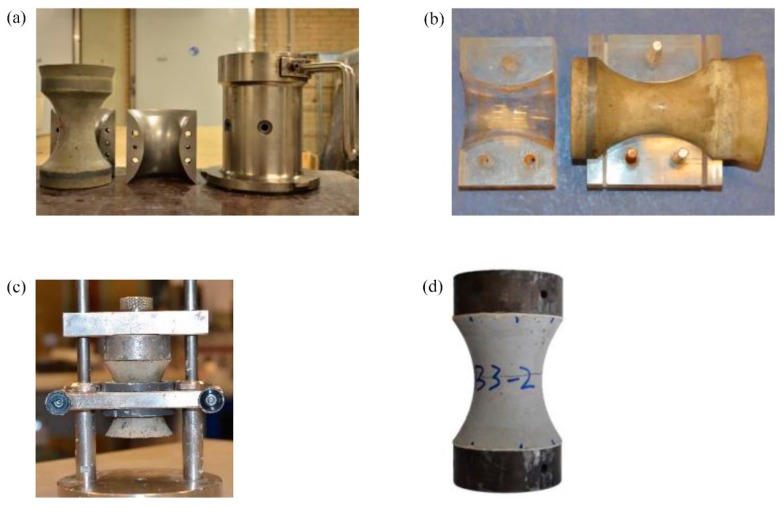
Preparation procedures of the parabolic-shape concrete specimen. (**a**) the parabolic specimen casted through a splitting mould inside the gyratory mould; (**b**) the Perspex cutting mould; (**c**) the gluing assistive device; (**d**) the specimen with steel caps.

This parabolic-shaped specimen is cast by an additional splitting mold inside the standard gyratory mold that is commonly used in asphalt concrete research, as shown in Figure 2a. The diameter of gyratory mold is 100 mm. The custom designed inner mold consists of two halves, which are assembled by connecting the two halves by screws. The specimen production is according to ASTM C192/C192M-07: “Standard Practice for Making and Curing Concrete Test Specimens in the Laboratory”. Because of the varying cross-section of the mold, sufficient compaction of the specimens is achieved by pre-compaction through manual rodding and subsequently placing the mold on an external vibrating table. No segregation has been found. After the compaction, the sample is cured at 20 °C and 100% relative humidity.

The specimen is demolded within 24 h from the time of mixing, and stored in a curing room at 20 °C and 100% relative humidity. On the pre-designated test day, the excess material outside the parabolic shape is cut off. To protect the specimens, especially the specimens tested at very early ages (1, 2, and 3 days) against being damaged during the cutting process, the specimen is placed in a cutting mold, as shown in Figure 2b. Another benefit of using the cutting mold is to make both cutting surfaces parallel to each other.

After cutting the excess material, the specimen is glued to the end caps that are used to transfer the load to the specimen in the uniaxial tensile test. Despite the use of the cutting mold, the acquired cutting surface can still be somewhat non-parallel, due to the slight movements of the cutting blade and some freedom in positioning it, especially when cutting the last portion of the excess material. Zhou [14] has concluded from numerical simulations that the load eccentricity has a significant effect on the softening behavior of concrete. To prevent gluing the caps in a non-parallel manner, a gluing mold is therefore used. The specimen is clamped at its center, using a PVC ring of 40 mm height that has the same shape as the concrete specimens, as shown in Figure 2c. This allows the glue to fill any gap that might exist between the cutting surface and cap. Two-component cold curing glue, X60, is used in the present study. Lastly, the glue used and gluing procedure have been proven very successful by the experimental results, as none of the specimens fractured at the specimen surface.

### 2.2. Test Set-Up

**Figure 3:**
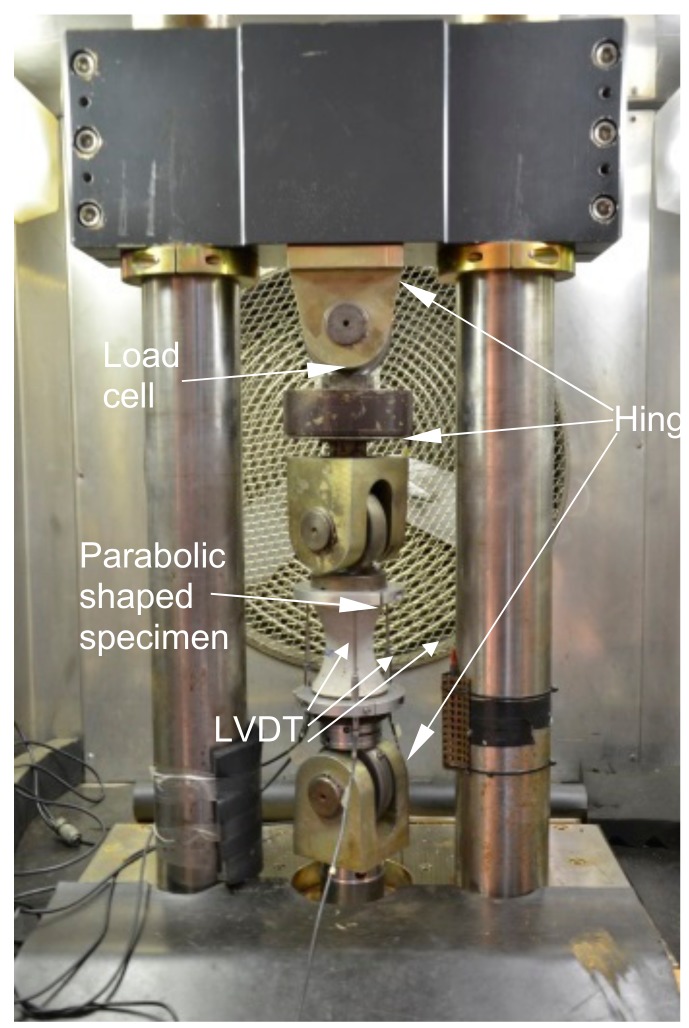
The TU Delft Uniaxial tensile test set up.

The TU Delft tension set-up with 3 hinges, developed as part of the Asphalt Concrete Response project [15], is used in this study, as shown in Figure 3. The key issue in a well-controlled uniaxial tension test of concrete is the influence of the specimen boundary conditions, however, this is still a matter of some debate in the literature [6,12]. Three types of boundary rotation conditions are generally applied in uniaxial tensile experiments: fixed, free, and mixed (the specimen is in between a fixed and a rotating plate). The last type of boundary condition is not very favorable, because the state of stress is rather complex. Under fixed boundary conditions, the experiment initiates a crack opening from the weakest side of the specimen, but because the specimen boundaries are forced to remain parallel during crack propagation, a closing bending moment develops. The descending branch of the force-deformation curve under fixed boundary conditions shows irregularities [12,16]. Among the free rotatable boundary conditions, two hinges (one at either side of the specimen) are used by most investigators. Ideally, this would be sufficient to ensure that the force is applied along the specimen’s axis, but in reality, the system may not be flexible enough to accommodate imperfect specimens, such as non-parallel surfaces. Two hinges give rotational freedom once a crack occurs, but they do not compensate for slight misalignments.

To compensate for misalignment, the TU Delft tension set-up introduced a third hinge, which is able to move horizontally to accommodate possible small deviations [15]. The actuator is connected rigidly to the bottom plate. A load cell is positioned underneath the upper hinge, and immediately below the load cell is the middle hinge. The lower hinge is placed on the actuator. The specimen is connected between the middle and lower hinges. In order to achieve a stable deformation-controlled uniaxial tensile test, the energy required for crack growth must remain larger than the energy released by the unloading of both the uncracked parts of the specimen and the loading frame [17]. If that is not the case, the crack will grow explosively, resulting in a vertical unloading branch or even a snap-back [16]. In this study, the tension set-up consists of a rigid loading frame, as shown in Figure 3, where the vertical bars are 100 mm in diameter and made of stainless steel to limit the energy stored in the frame.

In the present study, there is no straight part of the parabolic surface specimen. Thus, the full specimen length of 90 mm is used as the control length for the concrete specimen. Three linear variable differential transformers (LVDT) with a measurement length of 90 mm are installed at 120° intervals. One type of LVDT, Solartron AG 1, with a measuring capacity of ±1 mm, is used. The connection of the LVDTs to the caps is by means of a set of rings. One ring has three holes at 120° intervals in which the LVDTs are clamped, while another is a massive ring on which the LVDTs are placed. After the specimen is placed between the lower two hinges and instrumented with LVDTs, a small pre-load (approximately 0.2 kN) is applied to ensure that there is no play in the setup components, more specifically, the hinges.

### 2.3. Test Conditions

In order to study the evolution of the concrete properties during hardening, the uniaxial tensile tests are performed at different concrete ages: 1, 2, 3, 5, 7, 14, 28, and 90 days for each mixture. All the specimens are cured at 20 °C and 100% relative humidity. The average axial deformations measured with the 3 LVDTs were used as the feedback control signal for test control. The applied deformation rate that was much lower than previous reports of uniaxial tension tests for concrete, as it was 0.1 μm/s for the specimens older than 3 days [18]. However, specimens younger than 3 days suddenly broke just after the peak load under this deformation rate. Thus, for these specimens, the deformation rate was reduced to 0.05 μm/s. Due to space limitations, for more details about the test conditions, reference is made to [19].

## 3. Results

**Figure 4:**
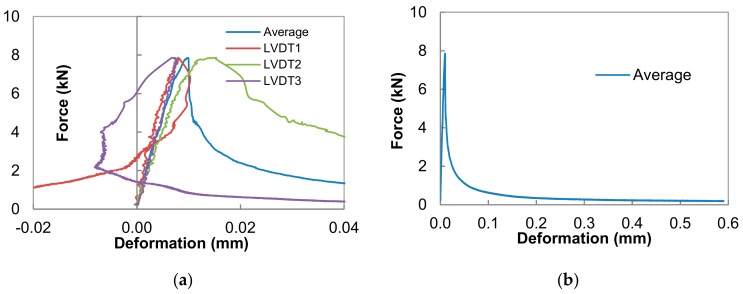
Overview of force-deformation curves for a specimen with finer aggregate at an age of 5 days. (**a**) The initial part of deformation-force curve for each LVDT; (**b**) the average deformation-force curves.

Figure 4a presents the raw experimental data of a specimen type 6.3 at an age of 5 days. In the pre-peak regime, the specimen behaves linearly, and is elastic up to 50% of the peak load, as shown by the measured deformation of all three LVDTs and the measured force. Furthermore, the curves become non-linear due to permanent plastic deformation until they reach the peak force. Just before the peak, LVDT number 1 and LVDT number 2 give a positive deformation and the largest deformation is measured with LVDT number 2. This indicates that the fracture initiates near LVDT number 2. At the other side of the specimen, near LVDT number 3, a compressive deformation (negative) is measured. When the total deformation increases, the crack grows rapidly in the direction of the LVDT number 3. Thus, the trend reverses: the tensile deformation develops between LVDT number 2 and 3, while the deformation near LVDT number 1 changes from tensile to compressive. The uniaxial tension tests were not continued until the complete separation of the two specimen halves. Because of the applied free rotation boundary conditions, the crack opening is non-uniform along the perimeter of the specimen, with tensile deformation at one side while compressive deformation develops on the opposite side of the specimen. Moreover, the average measured deformation of the three LVDTs is used as control signal. Due to the limited measuring range of the type of LVDT used, and the Solartron AG 1 with a measuring capacity of ±1 mm, one or even more LVDTs may reach their range when the total average deformation exceeds 0.6 mm. Thus, the average measured deformation is no longer adequate after one or more LVDT reach their range. As shown in Figure 4b, the descending branch of the average measured deformation approaches the horizontal axis very gradually. Most of the experiments were stopped at an average deformation of 0.6 mm in the present study.

**Figure 5:**
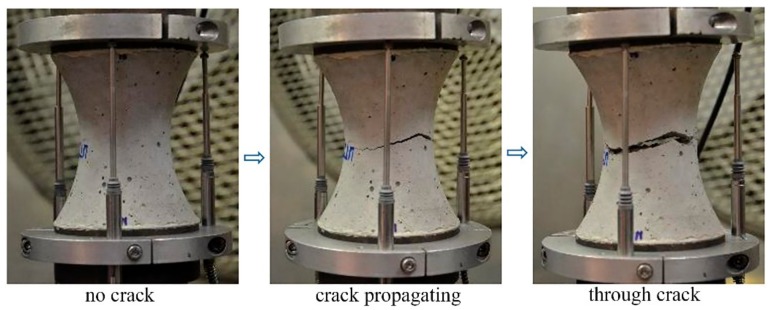
Crack development during a deformation controlled uniaxial tensile test.

**Figure 6:**
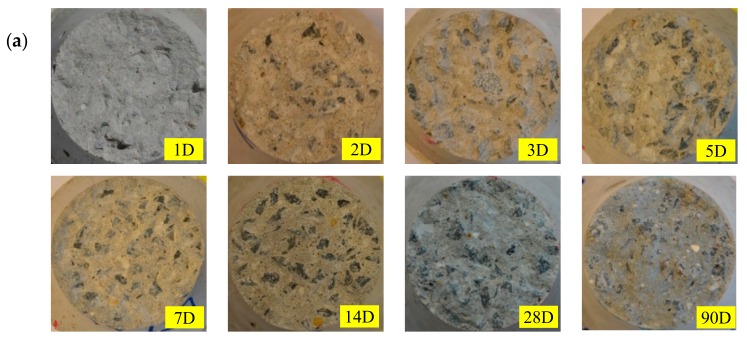

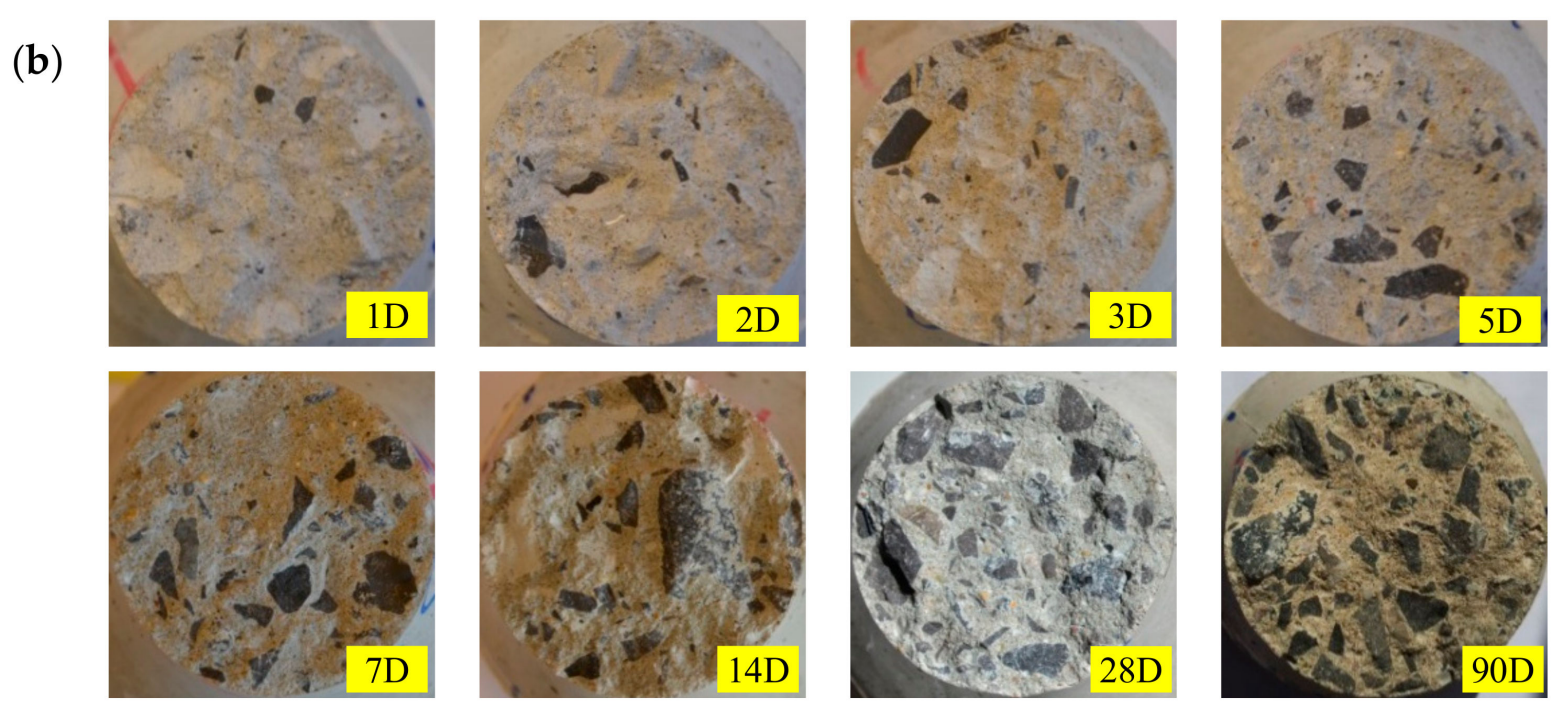
Fracture surface of specimens tested at increasing ages, (**a**) for specimen type 6.3; (**b**) for specimen type 16.

Figure 5 shows the crack propagation process of a specimen under rotating boundary conditions, conducted by the deformation controlled uniaxial tensile test in this study. The specimen starts to crack on one side, and propagates further into the same side until the crack develops entirely through the specimen. Moreover, it clearly indicates that the crack surfaces are not parallel when the crack is growing under the free boundary conditions. The fracture surface is also changing significantly with the age of the concrete and the type of the aggregate, as shown in Figure 6. At an early age, for instance 24 h, absolutely no aggregates are fracturing, which makes the fracture surface very rough, especially for the specimen type 16 with coarser aggregate. The fracture surface is much smoother at later ages, when the cement paste hardens.

**Figure 7:**
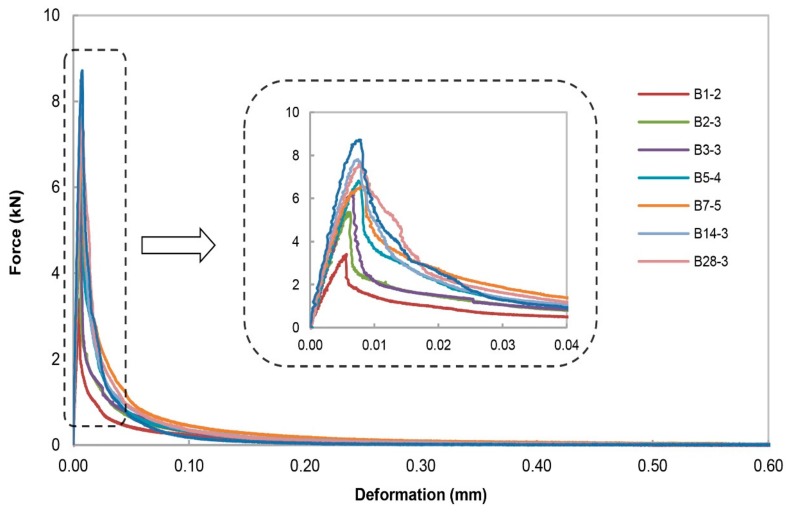
Overview of the force-deformation curves for specimen type 6.3 that were fractured at the center of the specimen as a function of testing age. ‘B1’ represents the specimen type 6.3 tested at an age of 1 day after casting; ‘-2’ represents the order number of the specimen for each specific age.

**Figure 8:**
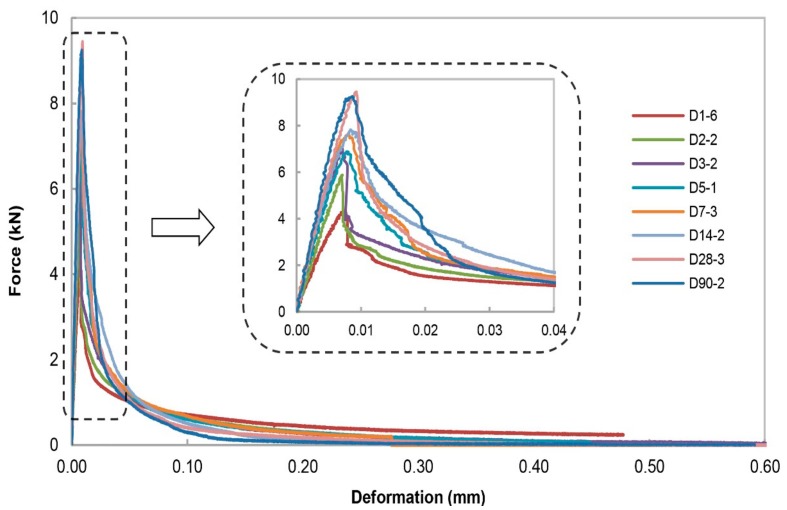
Overview of the force-deformation curves for specimen type 16 that were fractured at the center of the specimen as function of testing age. ‘D1’ represents the specimen type 16 tested at an age of 1 day after casting; ‘-6’ represents the order number of the specimen for each specific age.

Figure 7 and Figure 8 present the measured force-deformation curves for the specimen type 6.3 and the specimen type 16 at each individual testing age, respectively. Stable crack growth for all the specimens has been achieved. The test results suggest that the peak load increases with the increasing age of specimens. The curves indicate that the failure load and the slope of the elastic part of each specimen increase with age. Increase in the elastic slope can be attributed to the evolution of the Young’s modulus with time. The rapid fall of the curve after the peak load shows that concrete is a brittle material and that it becomes more brittle when it ages. Among the maximum aggregate size, the peak load for the specimen type 6.3 is found to be slightly larger than that for specimen with coarser aggregate size at the same testing age.

### 3.1. Correction of Experimental Data

#### 3.1.1. Deformation Data

In the pre-peak region, the measured deformation includes three components: specimen, caps, and glue layers. It has been found that the higher the concrete modulus and the thicker the glue layer, the smaller the percentage of specimen deformation to the measured overall deformation. For a matured concrete having a modulus of 40,000 MPa, 1.5 mm thick glue layers, and 30 mm of measuring distance on the steel caps, the deformations of the steel caps and glue account for 14.8% of the measured deformation by the LVDTs [19]. Thus, it clearly shows that the influence of the caps and the glue on the measurement cannot be considered negligible. The raw experimental deformation data therefore has to be corrected.

#### 3.1.2. Force Data

The load is measured by a load cell that was placed between the top hinge and the middle hinge, as shown in Figure 3. Before the middle hinge and the specimen with loading caps were placed in the experimental set-up, the load was calibrated to zero. Thus, the weight of the middle hinge, the top loading cap, and the weight of the specimen above the crack are included in the external force recorded by the load cell. Based on the force equilibrium in the vertical direction, the applied tensile force $F_{cr}$ on the fracture surface is determined by subtracting the sum of the weight of the middle hinge, the top cap, and the upper half of the concrete specimen from the measured force $F_{m}$ by the load cell. The self-weight term of 0.15 kN has been found for almost all the tested specimens in the present study. It is equal to a stress of 0.08 MPa, that is, around 3% of the concrete tensile strength during the hardening phase. Previous research has pointed out that the value of the fracture energy $G_{f}$ is very sensitive to an incorrect load measurement: an error of the zero load equal to 0.03 MPa leading to a 10% error of the determined fracture energy was reported by Hordijk [16].

#### 3.1.3. Correction for the F-δ Relation

**Figure 9:**
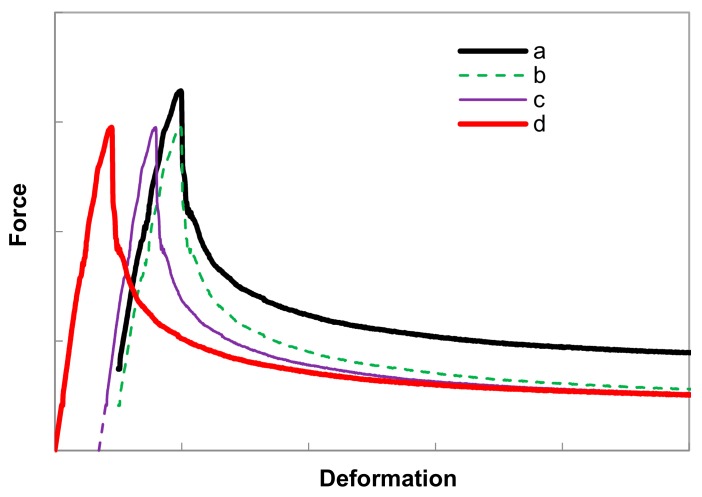
Schematic view of corrections made on the experimental data.

The raw experimental *F-δ* relation of a specimen with an age of 5 days is illustrated in curve *a* in Figure 9. Due to the flexibility of the hinges, the origin of the *F-δ* relation could not be determined by a compression test in which a deformation at zero load level is used to correct the deformation. In the present study, the origin of the *F-δ* relation is retrieved through back analysis. Firstly, the deformation of the specimen has been corrected though subtracting the deformation of the caps and the glue layers from the raw experimental data (curve *b*). Similarly, the external force applied on the fracture face has also been corrected by excluding the self-weight. The initial slope of the corrected curve (curve *c*) is linearly extrapolated until the intersection point with the horizontal axis of the deformation. Lastly, this intercept deformation value is then used to define the corrected *F-δ* relation through the origin (curve *d*).

### 3.2. Concrete Behaviour in a Deformation Controlled Uniaxial Tensile Test

**Figure 10:**
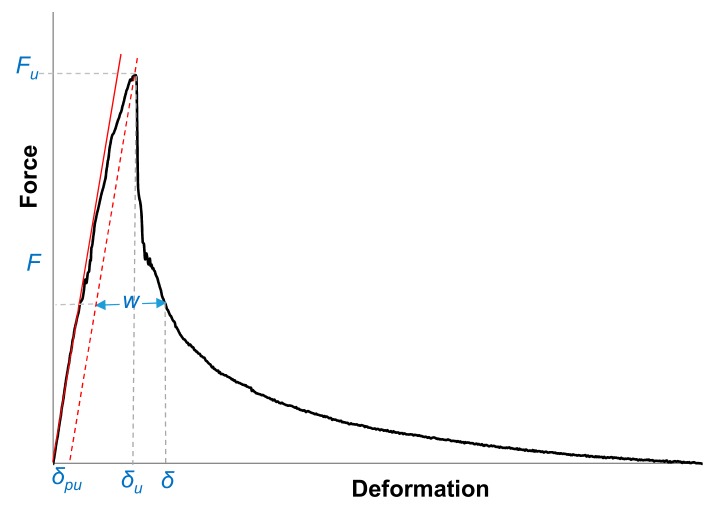
Schematic view of deformation components for a complete deformation controlled uniaxial tensile test.

As shown in Figure 10, in the pre-peak regime, a linear force-deformation relation up to half of the peak load is obtained. At the peak load, the strains start to localize within a narrow zone of micro-cracks at the weakest section of the tensile specimen. After that, a macro-crack will develop. The total deformation of the specimen is composed of three components namely, instantaneous elastic, visco-elastic, and permanent plastic deformation. Among those, the viscous elastic deformation for pavement concrete materials can be considered negligible. In addition, the measured deformation includes the crack opening, as well in the post-crack regime.

At the peak load:

(1) $$\delta_{u}=\delta_{pu}+\overset{h_{specimen}}{\underset{0}{\int }}\frac{F_{u}}{E_{c}A_{z}}dh$$

In the post-peak regime:

(2) $$\delta =\delta_{pu}+\overset{h_{specimen}}{\underset{0}{\int }}\frac{F}{E_{c}A_{z}}dh+w$$

where,

δ = the corrected average overall deformation of the specimen measured by three LVDTs, (mm);

$\delta_{e}$ = elastic deformation of the specimen under load level F, (mm);

$\delta_{p}$ = plastic deformation of the specimen under load level F in the pre-peak regime, (mm);

$\delta_{u}$ = the corrected ultimate measured overall deformation of the specimen at the peak load, (mm);

$\delta_{pu}$ = plastic deformation of the specimen under peak load $F_{u}$, (mm);

w = crack width, (mm);

F = the corrected load on the fracture surface, (N);

$F_{u}$ = the corrected peak load on the fracture surface, (N);

$E_{c}$ = the calculated elastic modulus of concrete in the pre-peak regime, (MPa).

The Equations (1) and (2) are put into Equation (3). Thus, the crack opening w is calculated as follows:

(3) $$w=\delta -\left(\delta_{u}-\overset{h_{specimen}}{\underset{0}{\int }}\frac{F_{u}}{E_{c}A_{z}}dh\right)-\overset{h_{specimen}}{\underset{0}{\int }}\frac{F}{E_{c}A_{z}}d$$

### 3.3. Determination of Fracture Energy

In a deformation controlled uniaxial tensile test on concrete, the fracture energy $G_{f}$ is calculated as the applied work of energy per unit fracture area, and it can be illustrated as:

(4) $$G_{f}=\frac{W_{F}}{A_{C}}=\frac{\int_{0}^{w_{c}}F_{cr}dw}{A_{C}}$$

where $W_{F}$ (N/mm) is the work of the fracture that is defined as the area under the post-peak of force-crack opening diagram. $F_{cr}$ (N) is the applied external force on the fracture surface of the concrete specimen, $A_{c}$ (mm^2^) is the area of the fracture surface considering the tortuosity of the fracture surface. Lastly, w and $w_{c}$ (mm) are the crack opening of the fracture surface and the critical crack opening at which stress is no longer transferred, respectively.

Previous researchers have pointed out several problems in the determination of fracture energy according to three-point bending tests on notched beams [20,21,22]. The first problem is to determine the critical crack opening $w_{c}$ at which stress is no longer transferred. Generally, the uniaxial tensile tests were not continued until complete separation of two halves. Most of the experiments were stopped at an average deformation of 0.6 mm in the present study. The measured deformation in the descending branch approaches the horizontal axis very gradually, mainly due to the capacity of selected LVDTs. One possible explanation of the long tail of the force-deformation curve might be a ‘hinge mechanism’, due to aggregates interlocking, which does not contribute to the fracture energy [23]. In the present study, a unique value $w_{c}$ = 0.16 mm is chosen according to the previous studies [24,25,26], which correlates the critical crack opening displacement with the maximum aggregate size. Thus, the corresponding calculated fracture energy is defined as $G_{f160}$. Apart from the above-chosen critical crack opening, the fracture energy until a crack width of about 0.6 mm is calculated as well, called $G_{f600}$.

Another problem in the determination of the fracture energy is the real area of the fracture surface. The area of the projected fracture plane is considered to be underestimating the true area of the fracture surface [27]. This is because of the tortuosity of the crack propagation path. More specifically, more tortuosity fracture surfaces were found for the younger age concrete specimens and the specimens with coarser aggregate [19]. The underestimated fracture surface area will lead to overestimation of the corresponding tensile strength and fracture energy. Therefore, a fracture surface correction coefficient $\alpha_{afs}$ for the area of the fracture surface introduced by Nallathambi and Karihaloo [27] is used in the present study. A $\alpha_{afs}$ = 1.1 was recommended based on their experimental data of the three-point bending tests on notched specimens.

Table 2 shows the significant influence of the selected values of critical crack opening on the calculated fracture energy. It can been seen that the calculated energy $G_{f600}$ is much higher than $G_{f160}$, and the largest difference was observed for the younger concrete. The data indicate that fracture energy increases with age in the period of 1 day to 90 days for both mixes. The fracture energy increased more rapidly within the first 3 days, and there is no apparent change in measured fracture energy when concrete matures. For the influence of aggregate size on fracture energy, a higher fracture energy is observed for the specimen type 6 (smaller aggregates), which does not coincide with the tendency from the literature. Moreover, a larger scattering of the measured results is observed for specimen type 16, which has an aspect ratio of 3.125. This may indicate that this aspect ratio is too small, which would give an insufficiently representative volume element to determine the fracture energy through this parabolic-shaped specimen. Hillerborg [28] concluded that the acceptance error and standard deviation of a fracture energy $G_{F}$ test is about 3 times larger than in most strength tests.

## 4. Discussion

### 4.1. Degree of Hydration-Based Concrete Mechnical Properites

In order to evaluate the risk of thermal cracking in a hardening concrete pavement, adequate descriptions of the time-dependent concrete properties are extremely important. However, the early age concrete mechanical properties commonly obtained through laboratory-cured specimens under relative constant curing conditions do not accurately represent the properties of field-placed concrete that experiences varying temperatures. The degree of hydration is considered to be a very fundamental parameter, therefore, the degree of hydration-based formulations of early age concrete mechanical properties can be considered more applicable to describe the evolution of hardening concrete under field conditions then time-based formulations [29].

In the present study, a degree of hydration-based formulation was calculated as a fraction of heat released to describe early age concrete mechanical properties.

(5) $$\frac{f_{t}\left(\alpha_{h}\left(t\right)\right)}{f_{t}\left(\alpha_{hmax}\right)}={\left(\frac{\alpha_{h}\left(t\right)-\alpha_{0}}{\alpha_{hmax}-\alpha_{0}}\right)}^{a}$$

(6) $$\frac{E_{c}\left(\alpha_{h}\left(t\right)\right)}{E_{c}\left(\alpha_{hmax}\right)}={\left(\frac{\alpha_{h}\left(t\right)-\alpha_{0}}{\alpha_{hmax}-\alpha_{0}}\right)}^{b}$$

(7) $$\frac{G_{f}\left(\alpha_{h}\left(t\right)\right)}{G_{f}\left(\alpha_{hmax}\right)}={\left(\frac{\alpha_{h}\left(t\right)-\alpha_{0}}{\alpha_{hmax}-\alpha_{0}}\right)}^{c}$$

where, $f_{t}\left(\alpha_{h}\left(t\right)\right)$, $E_{c}\left(\alpha_{h}\left(t\right)\right)$, and $G_{f}\left(\alpha_{h}\left(t\right)\right)$ are the degree of hydration-based uniaxial tensile strength, the elasticity modulus, and the fracture energy at the age t, respectively. $\alpha_{h}\left(t\right)$ is the degree of hydration of cement and $\alpha_{hmax}$ is the ultimate degree of hydration depending on the water cement ratio. $\alpha_{0}$ is the critical degree of hydration when the strength and stiffness start to develop. The degree of hydration $\alpha_{h}\left(t\right)$ of the laboratory-cured concrete specimens for each tested age was calculated as a fraction of heat released [18]. The coefficients a, b, and c are obtained by fitting the test results of deformation controlled uniaxial tensile tests.

Table 3 lists the mean values of the measured early age concrete mechanical properties for both mixtures. The nominal tensile strength $f_{t}$ is defined as the recorded maximum tensile load $F_{u}$ divided by the smallest cross section area of the specimen. The modulus of elasticity of concrete *Ec* under tension is calculated according to CRD-C 166 (1992): “Standard Test Method for Static Modulus of Elasticity of Concrete in Tension”. The chord modulus of elasticity is used as the modulus of elasticity, which is the slope of the chord drawn between 10% and 50% of the ultimate load on the pre-peak force-deformation curve [19]. The average values of the test results at 28 days are assumed to be the ultimate concrete properties at the end of cement hydration that can be achieved in practice. The regressed coefficients *a, b, c*, and the ultimate values at the end of the reaction are summarized in Table 4.

### 4.2. Development of Early Age Concrete Tensile Properites

According to the regression analysis of the calculated fracture energy in the present study, the value of coefficient *c* is smallest compared to that for tensile strength and elastic modulus, which indicates that the fracture energy develops much faster than the tensile strength and the elastic modulus of concrete. The present experimental results tend to confirm the tendency found in a few earlier studies [4,5,6,8]. Gaedicke et al. [8] observed that the evolution of fracture toughness, through wedge splitting tests of two mixtures for concrete pavements, was faster than that of the elastic modulus from 6 h to 24 h. Zollinger et al. [3] proposed a relation about the development of the fracture toughness of concrete as a function of the age of the specimen, $K_{If}/K_{If}^{28}={\left(t/28\right)}^{0.25}$, based on the size effect law for the three-point bending tests of various size notched beams with similar geometry.

**Figure 11:**
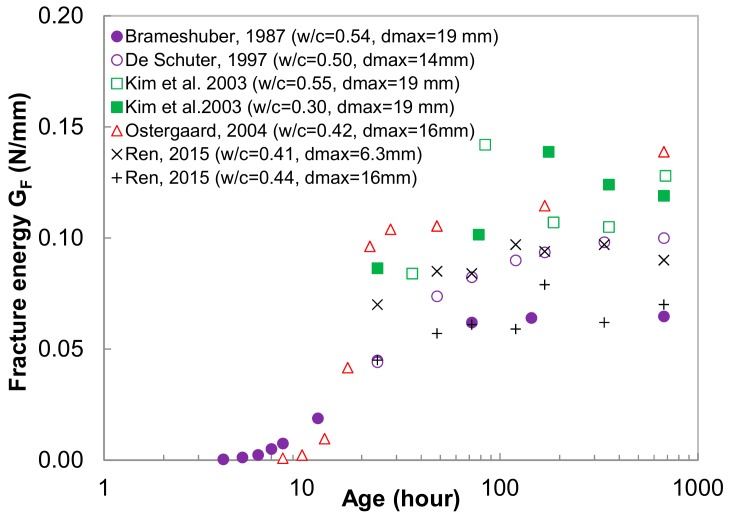
Summary of experimentally obtained early age concrete fracture energy for normal concrete.

Very few experimental data were reported on the fracture properties of early age concrete. A summary of these data are shown in Figure 11. The fracture energy of concrete before the final set is negligible and it increases very rapidly afterwards, which is similar to the development of the tensile strength of concrete. Moreover, there is a wide scattering of measured fracture energy at 28 days, due to different concrete compositions and various testing methods. The measured fracture energy in the present study is lower than the others. This is mainly due to the cement type used in the current study. Schutter and Taerwe [5] had found that blast-furnace cements have a lower rate of development and a lower final value than ordinary Portland cement.

## 5. Conclusions

The tension set-up developed in the present study worked very well for obtaining the fracture energy of concrete at early age. Due to the adopted small specimen size, very fast servo system, and stiff testing frame, a stable experiment and a complete softening curve were obtained, even for concrete specimens with an age as early as 24 h. Based on an extensive deformation controlled uniaxial tensile test program on two typical pavement concrete mixtures used in Belgium, the following conclusions can be drawn:

- The applied parabolic shape of the concrete specimens ensured that the crack occurred near the center of the specimen, where it was in the intended state of more or less uniform stress. Moreover, this parabolic-shaped specimen, with a considerable reduction of cross-section area (nearly 60%) at mid-height, let the cracks initiate far enough from the end caps to prevent the influence of confinement there.
- The loading rate of 0.1 μm/s, corresponding to a measuring length of 90 mm, proved successful in preventing the crack from growing explosively for concrete with an age greater than 48 h, as no snap-back was observed in the post-peak region of all the measured force-deformation curves. A lower loading rate of 0.05 μm/s was subsequently found to provide a stable complete uniaxial tension test for concrete with an age of 24 h.
- The fracture energy, uniaxial tensile strength, and elasticity modulus were all found to increase with age, going towards a horizontal asymptote as concrete hardened in a tested age range of 1 day to 90 days. More work is needed to identify factors such as aggregate size, water to cement ratio, loading rate, etc., that affect the tensile properties of very early age concrete.
- Gluing the specimen to the loading plates may be impossible on a very early age concrete specimen, and the proposed tension test set-up and test procedures in the present study might be not suited for the very early age concrete (less than 24 h). An indirect method, such as a horizontal tensile test, might be an alternative option for obtaining softening curves for very early age concrete.

## Figures and Tables

**Table 1:** Concrete Mixture Composition.

Parameter	Unit	E17	E313	
			Top	Bottom
Cement	kg/m^3^	400	425	375
Water	kg/m^3^	172	175	165
Water to cement	-	0.43	0.41	0.44
Coarse aggregate	kg/m^3^	1331	1030	1149
Coarse aggregate type	-	porphyry	porphyry	limestone
Fine aggregate	kg/m^3^	474	664	726
Air-entrainer	kg/m^3^	0.43	0.43	0
Plasticizer	kg/m^3^	1.28	1.28	1.05
Air content	%	3	3	-

**Table 2:** Fracture energy (N/mm) vs. the age of the specimens.

Age (Day)			1	2	3	5	7	14	28	90
6.3	$G_{f160}$	Mean	0.070	0.085	0.084	0.097	0.094	0.097	0.090	0.093
		SDEV ^1^	0.014	0.011	0.006	0.016	0.014	0.009	0.011	0.007
		CV ^2^	19.79	13.24	7.48	16.78	15.29	8.80	11.88	7.95
	$G_{600}$	Mean	0.109	0.120	0.115	0.126	0.114	0.111	0.107	0.107
		SDEV	0.030	0.023	0.007	0.026	0.023	0.004	0.014	0.011
		CV	27.29	18.95	6.41	20.52	20.32	3.58	12.64	9.90
16	$G_{f160}$	Mean	0.045	0.057	0.061	0.059	0.079	0.062	0.070	0.064
		SDEV	0.011	0.006	0.004	0.006	0.016	0.010	0.009	0.012
		CV	23.70	10.67	6.66	10.16	19.89	16.15	12.70	18.93
	$G_{600}$	Mean	0.071	0.089	0.081	0.070	0.097	0.069	0.078	0.071
		SDEV	0.019	0.036	0.013	0.006	0.020	0.015	0.009	0.011
		CV	26.91	39.90	16.18	7.83	20.59	21.88	11.15	17.39

^1^ SDEV is the standard deviation of the calculated fracture energy in terms of each defined critical crack opening, and ^2^ CV is the corresponding coefficient of variation.

**Table 3:** Mean values of early age concrete properties vs. degree of hydration.

Age (Days)	Degree of Hydration (-)	$f_{t}\;(\mathrm{MPa})$		$E_{c}\;(\mathrm{MPa})$		$G_{f160}\;(N/\mathrm{mm})$	
		6.3	16	6.3	16	6.3	16
1	0.324	2.04	1.63	23,500	21,167	0.070	0.045
2	0.548	2.95	2.66	31,200	30,833	0.085	0.057
3	0.638	3.18	3.04	32,750	34,500	0.084	0.061
5	0.727	3.73	3.06	37,300	33,667	0.097	0.059
7	0.773	3.80	3.05	37,800	38,667	0.094	0.079
14	0.834	4.15	3.69	38,833	38,833	0.097	0.062
28	0.858	4.77	3.63	38,100	39,167	0.090	0.070

**Table 4:** Regression coefficients of degree of hydration based early age concrete fracture properties.

Properties	Specimen Type	
	6.3	16
$f_{t}\left(\alpha_{hmax}\right)$ (MPa)	4.77	3.63
*a* (-)	0.93	0.65
$E_{c}\left(\alpha_{hmax}\right)$ (MPa)	39,000	39,000
*b* (-)	0.40	0.48
$G_{f}\left(\alpha_{hmax}\right)$ (N/mm)	0.100	0.070
*c* (-)	0.31	0.37
